# The effect of traumatic brain injury on learning and memory: A synaptic focus

**DOI:** 10.1177/10738584241275583

**Published:** 2024-09-24

**Authors:** Eric Eyolfson, Kirsten R. B. Suesser, Holly Henry, Itziar Bonilla-Del Río, Pedro Grandes, Richelle Mychasiuk, Brian R. Christie

**Affiliations:** 1 Division of Medical Sciences and Institute for Aging and Lifelong Health, University of Victoria, Victoria, BC, Canada; 2 Department of Neurosciences, Faculty of Medicine and Nursing, University of the Basque Country, Leioa, Spain; 3 Achucarro Basque Center for Neuroscience, Science Park of the University of the Basque Country, Leioa, Spain; 4 Department of Neuroscience, Central Clinical School, Monash University, Melbourne, VIC, Australia; 5 Island Medical Program and Department of Cellular and Physiological Sciences, University of British Columbia, Vancouver, BC, Canada; 6 Department of Psychology, San Diego State University, San Diego, CA, USA

**Keywords:** synaptic plasticity, long-term potentiation, long-term depression, endocannabinoids, dentate gyrus, hippocampus, electrophysiology

## Abstract

Deficits in learning and memory are some of the most commonly reported symptoms following a traumatic brain injury (TBI). We will examine whether the neural basis of these deficits stems from alterations to bidirectional synaptic plasticity within the hippocampus. Although the CA1 subregion of the hippocampus has been a focus of TBI research, the dentate gyrus should also be given attention as it exhibits a unique ability for adult neurogenesis, a process highly susceptible to TBI-induced damage. This review examines our current understanding of how TBI results in deficits in synaptic plasticity, as well as how TBI-induced changes in endocannabinoid (eCB) systems may drive these changes. Through the synthesis and amalgamation of existing data, we propose a possible mechanism for eCB-mediated recovery in synaptic plasticity deficits. This hypothesis is based on the plausible roles of CB1 receptors in regulating inhibitory tone, influencing astrocytes and microglia, and modulating glutamate release. Dysregulation of the eCBs may be responsible for deficits in synaptic plasticity and learning following TBI. Taken together, the existing evidence indicates eCBs may contribute to TBI manifestation, pathogenesis, and recovery, but it also suggests there may be a therapeutic role for the eCB system in TBI.

Traumatic brain injury (TBI) is a significant health issue worldwide (Dams-O’Connor and others 2023; Rivera-Lara and others 2023). TBIs can be caused by the physical forces imparted on the brain by a plethora of events that can include motor vehicle accidents, sports-related collisions, falls, military duty, and intimate partner violence (Dow-Fleisner and others 2023; Maldonado-Rodriguez and others 2021; Smirl and others 2019). Because the origin of TBIs can be so varied, the extent of the injury can range from being classified from mild to severe, but ~75% to 80% of all cases are classified as mild TBI (mTBI) (Harris and others 2024). TBI pathophysiology involves both primary and secondary injuries, and these often dictate the severity and type of neurologic impairment/damage observed at acute and chronic time points (for reviews on primary injuries, see Johnson and others 2013; for secondary injuries, see Barkhoudarian and others 2016; Giza and Hovda 2001). The prevalence of TBI also varies across age groups, being particularly important in pediatric and senior populations. Both clinical and preclinical models highlight the heterogeneity in TBI pathophysiology that is influenced by factors such as injury severity, age at injury (Mannix and others 2017), number of injuries (Wright and others 2017), interinjury interval (Longhi and others 2005), and biological sex (Covassin and others 2013; Covassin and others 2010; Covassin and others 2018; Covassin and others 2007; Eyolfson and others 2021; Kerr and others 2016; Wallace and others 2017). Because each TBI is unique, they can present with a varied array of symptomologies across multiple behavioral modalities. For the purpose of this review, we will focus on significant reductions in general cognitive ability, learning and memory, and short-term working memory (de Freitas Cardoso and others 2019). Critically, cognitive impairments are also associated with persistent postconcussive symptomologies that can be identified up to six months or a year postinjury (de Boussard and others 2005).

This is best exemplified in the hippocampal formation (HPC), a bilateral subcortical structure that plays a pivotal role in learning and emotional processes and is also prone to damage in mTBI (Pinar and others 2020). The human and rodent HPC are composed of two interlocking gyri called the hippocampus proper or cornu ammonis (CA) and the dentate gyrus (DG). The CA is composed of pyramidal cells, while the DG is composed of granule cells. The flow of information in the HPC is often described as a unidirectional “loop” (Pinar and others 2017). The external input comes from the entorhinal cortex, which projects via the performant path to primarily synapse onto DG granule cells (although direct synapses onto CA3 and CA1 pyramidal cells exist). In turn, the DG granule cells synapse onto the CA3 pyramidal cells as the mossy fiber pathway. The CA3 cells then synapse onto CA1 pyramidal cells via the Schaffer-collateral pathway (Andersen and others 1966; Stepan and others 2015).

Synaptic plasticity, or the capacity for synapses to alter their functional connectivity, remains the preeminent model for learning and memory processes in the central nervous system (CNS). Long-term potentiation (LTP) of synaptic plasticity refers to an increase in synaptic connectivity, while long-term depression (LTD) refers to a decrease. Either one or both of these processes can be affected by external forces (Fontaine and others 2019). For example, the juvenile brain exhibits an increased capacity for LTP but not LTD in response to physical exercise (Vasuta and others 2007). However, not all experiences affect synaptic plasticity in all regions of the HPC equally (Anvari and others 2023; Dahlin and others 2019; Schreurs and others 2017; Van Praag and others 1999a; Wang and others 2016; White and others 2017; for review, see Alkadhi 2019). In some circumstances, one hippocampal subfield, such as the DG, may be affected more than others. The DG subfield is composed of granule cells that receive input from the entorhinal cortex. Unlike the CA1 region, studies of synaptic plasticity in this region in vitro require the use of a GABA-A antagonist as the perforant path synapses also synapse onto inhibitory cells that are directly activated when the stimulating electrodes are located in the molecular layer (Wigström and Gustafsson 1985). While it is well established that moderate-to-severe TBI affects the capacity for synaptic plasticity within the Schaffer collateral inputs to the CA1 of the HPC (Albensi and others 2000; D’Ambrosio and others 1998; Norris and Scheff 2009; Reeves and others 1995; Schwarzbach and others 2006; Yaka and others 2007; Zhang and others 2011), research has often overlooked the DG, but it does appear that it can also be affected by mild TBI (White and others 2017). Given the structural and connectivity differences in the DG to the rest of the HPC, it is imperative that we adequately understand the synaptic plasticity deficits that occur following mTBI and how they are related to functional outcomes.

An interesting lens through which to explore synaptic plasticity changes following TBI is the endocannabinoid system. There are two primary cannabinoid receptors. CB1Rs are primarily located on presynaptic terminals in the CNS and vary based on brain region and age (Yoshida and others 2006). Conversely, CB2Rs are primarily expressed by immune cells in the brain, including microglia, which synthesize and release endogenous cannabinoids (eCBs) (Carrier and others 2004). Interestingly, the hippocampus has a high concentration of CB1Rs (primarily on inhibitory terminals but also present on excitatory terminals, mitochondria, dendrites, and other membranes), and activation of CB1Rs has been shown to impair hippocampal-dependent learning tasks (Bonilla-Del Río and others 2021; da Cruz and others 2020; Loureiro and others 2015; Peñasco and others 2019). CB receptors can be activated by both exogenous plant-based cannabinoids (tetrahydrocannabinol [THC]; cannabidiol [CBD]) and by eCBs that include anandamide (AEA) and 2-arachidonoyl glycerol (2-AG). These eCB molecules seem to normally function as retrograde messengers that are released by postsynaptic cells in response to depolarization. They bind to CB receptors on presynaptic terminals, where they inhibit transmitter release at both excitatory and inhibitory synapses (Devane and Axelrod 1994; Ohno-Shosaku and others 2002). It has been shown that eCBs play a role in the induction of LTP (Silva-Cruz and others 2017; Wang and others 2016) and LTD (Fontaine and others 2020; Peñasco and others 2019). In the CA1 subfield, their role in LTP involves reducing presynaptic transmitter release (Misner and Sullivan 1999), but CB receptors may have other roles when located on nonneuronal cells. Reductions in synapse number have been reported following several preclinical mTBI models, and this could reflect altered activation of microglia (Eyolfson and others 2022; Krukowski and others 2018; Krukowski and others 2021; Ratliff and others 2020; for review, see Jamjoom and others 2021) or an increase in synapse elimination by LTD processes (Trivino-Paredes and others 2019). Thus, eCBs may affect synaptic plasticity after TBI either directly by affecting the induction of LTP and LTD or indirectly by altering synapse numbers and the functional capacity for synaptic plasticity.

Given that eCBs play an important role in the regulation of bidirectional synaptic plasticity, they are likely pivotal to our understanding of the mechanisms underlying synaptic plasticity in the context of learning and memory and TBI (Ferreira-Vieira and others 2014; Morena and others 2015; Varvel and others 2005). Cognitive and memory deficits often manifest following TBIs, resulting in issues with attention, episodic memory, working memory, and executive functioning (Dikmen and others 2009). Therefore, the purpose of this review is to synthesize and evaluate the recent advances in the mTBI field (also encompassing repetitive mTBI [r-mTBI]) regarding changes in synaptic plasticity. In particular, we propose that mTBI diminishes the influence of eCB signaling, leading to impaired bidirectional synaptic plasticity in the HPC. Thus, eCB signaling may be a key mechanism for understanding mTBI-induced deficits. Gaining a deeper understanding of the mechanistic basis of how synaptic plasticity is altered is essential for the development of novel therapies.

## Synaptic Transmission

For proper neural network functioning, a balance between synaptic inhibition and excitation must exist (Hartman and others 2006). Inhibitory synaptic transmission is regulated by y-aminobutyric acid (GABA) receptors, specifically the GABA-A subunit. GABA receptors are found ubiquitously throughout the hippocampus on interneurons and presynaptic terminals of excitatory neurons (for review, see Pelkey and others 2017; Tzilivaki and others 2023). GABA-A receptors are primarily responsible for hyperpolarization of excitatory neurons, allowing for a balance of excitation and inhibition (Palpagama and others 2019). GABAergic inhibition has been suggested to set the conditions for synaptic change in hippocampal neurons and can modulate the induction and expression of LTP and LTD (Paulsen and Moser 1998; Wigström and Gustafsson 1985). Excitatory synaptic transmission is driven primarily by glutamate receptors, such as *N*-methyl-d-aspartate (NMDA) receptors and α-amino-3-hydroxy-5-methyl-4-isoxazole propionate (AMPA) receptors, which are located primarily on dendritic spines (Zamanillo and others 1999). AMPA receptors (AMPARs) play a predominant role in excitatory glutamatergic neurotransmission, and the concentration and composition of these receptors determine the speed of glutamatergic signaling (Gan and others 2015). The cooperativity of these receptors is imperative for changes in synaptic strength, which is characterized by LTP and LTD. The mechanisms of LTP and LTD have been extensively reviewed elsewhere (Collingridge and others 2010; Lüscher and Malenka 2012; Malenka and Bear 2004), and as such, this review will give a broad overview of the processes and how they may relate to injury pathogenesis.

Long-term changes in synaptic strength occur in an activity-dependent manner, with multiple mechanisms existing to induce LTP and LTD. LTP involves high-frequency stimulation of excitatory synapses to produce a rapid and long-lasting increase in the strength of synapses that can persist for many days (Bliss and Gardner-Medwin 1973; Bliss and Lømo 1973). The time course of LTP induction and maintenance is divided into an initial phase (in the order of seconds), an early phase (in the order of minutes), and the consolidation phase (in the order of hours to days). LTP was first described in the hippocampus (Bliss and Lømo 1973), and perhaps the most well-documented form of LTP relies primarily on the activation of NMDA receptors by high-frequency stimulation (HFS). However, the induction of LTP is not equivalent throughout the hippocampus. For example, the use of a GABA-A antagonist, such as picrotoxin or bicuculline methiodide, is required for LTP induction in the DG but not CA1 (Wigström and Gustafsson 1983) (Box 1).

**Box 1.**
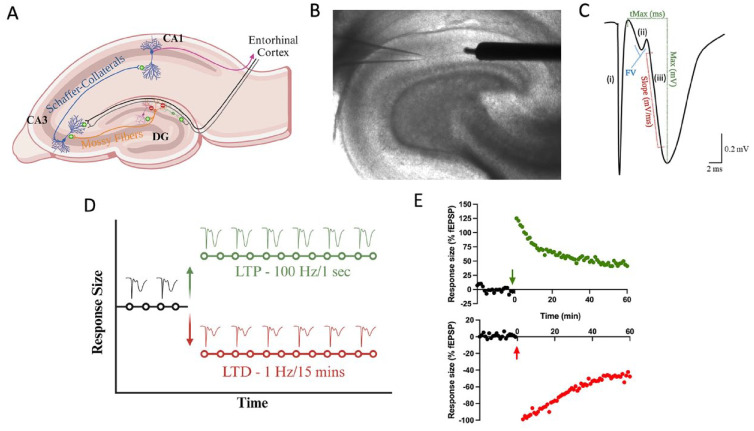
Introduction to field electrophysiology. (A) Major hippocampal pathways that compose the trisynaptic loop. The dentate gyrus receives both feed-forward and feed-back inhibition from local interneurons. In vitro electrophysiology necessitates a GABA-A antagonist to block feed-forward inhibition. Primary recording pathways for in vitro field electrophysiology include the medial and lateral performant pathways in the dentate gyrus and Schaffer collateral pathways in CA1. (B) Brightfield micrograph (4× magnification) of transverse hippocampal tissue recording in the medial perforant pathway of the dentate gyrus. It illustrates the placement of a concentric bipolar stimulating electrode (right) and glass recording pipette (left). (C) Sample waveform characteristic of in vitro field electrophysiology experiments: (i) Stimulation artifact, indicating the conduction of electricity through the artificial cerebral spinal fluid (aCSF) medium. (ii) Fiber volley, signifying presynaptic neurotransmitter release as electricity moves from the stimulating to the recording electrode. (iii) Presynaptic neurotransmitter release activates postsynaptic receptors, initiating a postsynaptic excitatory response, initially depolarized by AMPA receptors and subsequently NMDA receptors. To gauge synaptic communication strength, the initial slope of the field excitatory post-synaptic potential (fEPSP) is measured. (D) Graphical representation of the slope of the fEPSP denoted as response size across time. Stable baseline recordings (typically a minimum of 20 minutes) precede evoke responses. Following the baseline period, high-frequency stimulation (100 Hz/1 s) can induce an increase the size of the responses (known as long-term potentiation), while low-frequency stimulation (1 Hz/15 min) can decrease response size (known as long-term depression). (E) Sample traces of hippocampal slices subjected to high-frequency or low-frequency stimulation, presented as a percentage of the baseline recording. Each form of bidirectional synaptic plasticity exhibits a significant initial increase (+125% in this HFS induction) or decrease (–100% in this low-frequency stimulation induction) in response size that eventually stabilizes. The recording duration is generally 60 minutes following high- or low-frequency stimulation as the mechanisms for LTP and LTD transition into protein-dependent phases.

**Figure 1.**
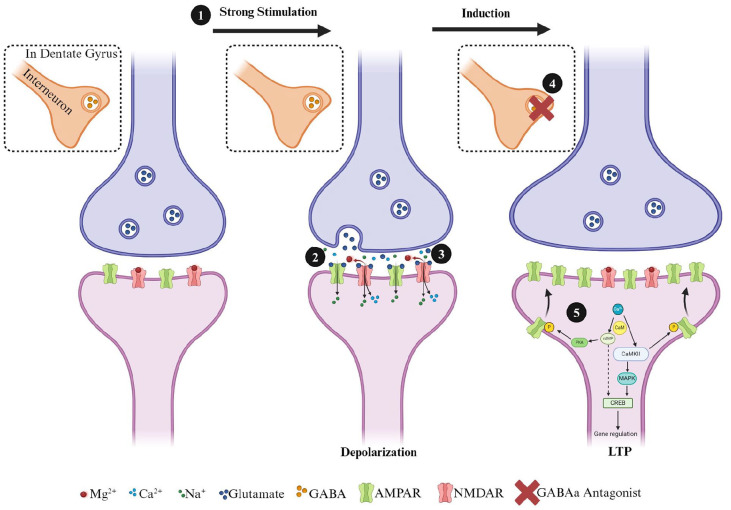
Simplified diagram of steps involved in the induction of a postsynaptically mediated form of long-term potentiation induction at hippocampal excitatory synapses. Excitatory glutamatergic synapses normally possess a complement of AMPA and NMDA receptors in the postsynaptic terminal. The administration of (1) high-frequency (i.e., 100 Hz) conditioning stimulation causes the release of glutamate (2), which primarily activates AMPARs initially (3). In the dentate gyrus (DG) (4), GABAergic synapses can also play a significant role in the induction of LTP in the medial perforant path synapses, and LTP induction normally requires blocking feedforward inhibition with GABA-A antagonists (i.e., bicuculine or picrotoxin). When inhibition is blocked, the activation of AMPARs results in an influx of Na^+^ into the postsynaptic neuron, depolarizing the synapse and alleviating the MG2^+^ block of the NMDAR pore. (5) The NMDARs can now influx Ca2^+^ into the postsynaptic cell, and this activates calmodulin (CaM) and calcium-calmodulin dependent protein kinase II (CamKII). CaM also activates adenyl cyclase, which results in the production of cyclic adenosine monophosphate (cAMP) and then activation of protein kinase A (PKA) by cAMP. Kinases, such as CamKII and PKA, phosphorylate GluRs, leading to the insertion of additional AMPARs into the postsynaptic membrane. CamKII and cAMP also activate mitogen-activated protein kinase A, which is translocated to the nucleus and activates cAMP response binding element-binding protein (CREB). CREB is a transcription factor and binds to the CRE response element, leading to the transcription of genes necessary for long-lasting protein synthesis-dependent forms of LTP.

HFS is required to depolarize the postsynaptic neuron sufficiently to remove Mg^2+^, which normally blocks NMDARs’ access to the glutamate binding site (Bliss and Collingridge 1993; Calabresi and others 1992; Collingridge and others 1983; Liu and others 2004; Lu and others 2001). With both postsynaptic depolarization and glutamate release, NMDARs allow an influx of Ca^2+^ into the postsynaptic neuron (Fig. 1). Through a series of subsequent secondary cascades with protein kinases, which can include phosphorylation of calcium/calmodulin-dependent kinase II (CamKII), AMPARs are translocated to the cell membrane via exocytosis (Lisman and Zhabotinsky 2001; Penn and others 2017). This results in not only changes in AMPAR number but also differences in the composition of subunits as well (Araki and others 2015; Boehm and others 2006; Heinze and Rust 2023). The exact mechanisms of AMPAR recruitment to the postsynaptic membrane are not fully understood, but it may be the result of amyloid precursor protein (Livingstone and others 2021), extrasynaptic surface AMPARs (Penn and others 2017), or cAMP-dependent protein kinase (Park and others 2021). Research has shown that protein expression of CamKII is hyper-acutely enhanced and subacutely reduced in the hippocampus following moderate-to-severe TBI. Additionally, there may be regional differences with greater reductions on CA1 and not the DG (Atkins and others 2006). The authors suggest this decrease in CamKII may be responsible for the diminished capacity to induce and maintain LTP (Schwarzbach and others 2006). CamKII is not the only kinase that can regulate changes in synaptic strength. Protein expression of phosphorylated PKA is reduced in the hippocampus four hours after moderate-to-severe TBI (Atkins and others 2007). Interestingly, a phosphodiesterase-4 inhibitor rescues LTP deficits following moderate-to-severe TBI as early as one day and as long as two weeks postinjury (Titus and others 2013; Wilson and others 2016). Additionally, treatment with the phosphodiesterase-4 inhibitor rescued TBI-induced deficits in spatial learning and fear conditioning (Titus and others 2013).

**Figure 2.**
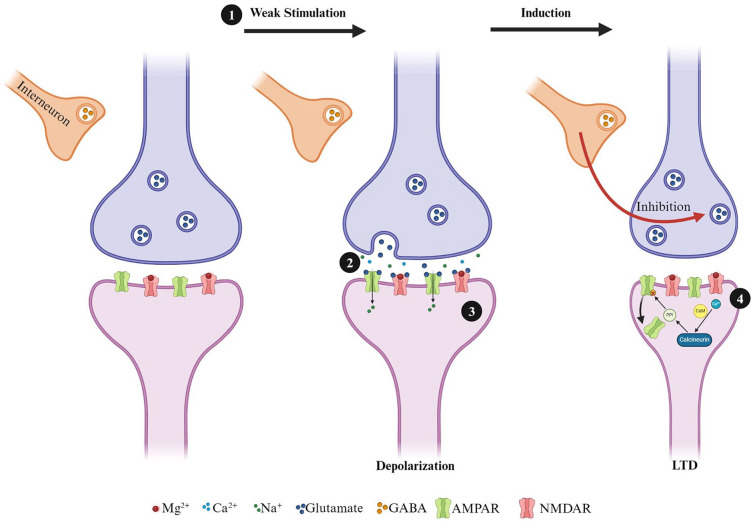
Simplified diagram of long-term depression induction in hippocampal excitatory synapses. (1) Induction of a low-frequency stimulation leads to modest depolarization, which (2) releases glutamate and activates AMPARs. (3) Activated AMPARs results in an influx of Na^+^ into the postsynaptic neuron but not enough to fully clear the MG2^+^ block of the NMDAR pore. (4) Influx of Ca2^+^ results in the activation of CaM, which leads to the activation of calcineurin. Calcineurin activates phosphatases such as protein phosphatase 1, leading to the dephosphorylation and endocytosis of AMPARs.

LTD is a complementary form of synaptic plasticity that reduces the strength of synaptic communication. In vitro, a homosynaptic form of LTD can be induced using prolonged low-frequency stimulation (Dudek and Bear 1992), although associative and heterosynaptic forms of LTD are more commonly observed in vivo (Pinar and others 2017). In addition, while low-frequency stimulation is commonly used in vivo to induce LTD in the hippocampus, its effectiveness and the mechanisms it engages are highly age dependent. For instance, numerous studies have shown that LTD in vitro can be NMDA dependent in young animals but NMDA independent in adult animals (Kemp and others 2000; Norris and others 1998). While LTP requires the insertion of AMPARs, LTD requires the removal of AMPARs via endocytosis (Carroll and others 1999). The induction of LTD can be triggered by the activation of NMDARs or metabotropic glutamate receptors (mGluRs). Although the signaling mechanisms for NMDA dependence are too complex to examine for this review, in brief, LTD involves Ca^2+^ entry through NMDARs (Fig. 2). Calmodulin detects Ca^2+^ and, through a cascade of events, signals protein phosphatase 1 (PP1). PP1 has an important functioning in dephosphorylating AMPARs (Yan and others 1999), PSD95 (Kim and others 2007), and glycogen synthase kinase-3B (GSK3B) (Peineau and others 2007). Finally, endocytosis of AMPAR receptors is thought to be the result of the release of internal Ca^2+^ stores (Beattie and others 2000). The maintenance of LTD can be the result of changes in the expression of PSD95 in the postsynaptic neuron, as well as alterations in the balance between AMPAR endocytosis and exocytosis (Fujii and others 2018). With respect to LTD and mTBI, the dysregulated activity of GSK3B in the hippocampus may be responsible for learning and memory deficits postinjury. In a recent study, adult CD-1 mice received a single closed-headed weight-drop injury and were administered an antisense oligonucleotide designed to target the GSK3B gene. Mice in the mTBI group, who received the antisense oligonucleotide, displayed improved memory in an object-place recognition and fear avoidance task up to four weeks postinjury (Farr and others 2019). Dysregulation of GSK3B may drive silencing of synapses, resulting in unabated synaptic pruning and cognitive deficits.

In addition to bidirectional changes in synaptic strength, the DG of the HPC is one of the few brain regions that maintains the ability to create new neurons (neurogenesis) throughout adulthood (for review, see Christie and Cameron 2006). In addition, adult neurogenesis can be highly influenced by environmental experience. For example, voluntary exercise and environmental enrichment across the life span enhance neurogenesis in the DG (Eadie and others 2005; Farmer and others 2004; Van Praag and others 1999b). The functional integration of newborn neurons can lead to changes in the functioning of the HPC. For example, in response to voluntary exercise, the juvenile brain exhibits an increased capacity for LTP, but not LTD, and these animals typically perform better on learning and memory tasks (Cameron and Glover 2015; Cameron and Mckay 2001; Snyder and others 2001; Van Praag and others 1999a; Vasuta and others 2007). While it is well established that moderate-to-severe TBI affects the capacity for synaptic plasticity within the CA1 of the HPC (Albensi and others 2000; D’Ambrosio and others 1998; Hung and others 2023; Norris and Scheff 2009; Reeves and others 1995; Schwarzbach and others 2006; Titus and others 2016; Yaka and others 2007; Zhang and others 2011), research has often overlooked the DG. Given the DG’s capacity for neurogenesis and its position in the HPC as the initial input for cortical information, this region of the hippocampus warrants further attention.

### Synaptic Plasticity in mTBI

**Figure 3.**
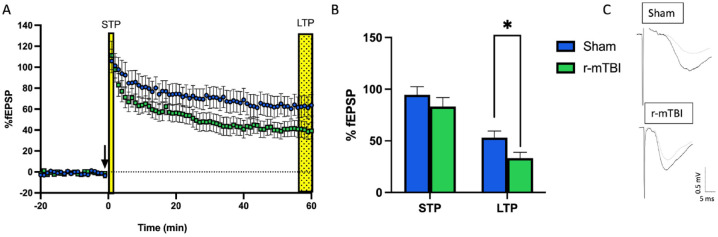
Deficits in long-term potentiation are seen in adolescent female animals following three repetitive injuries (72-hour interinjury interval) in the medial perforant pathway of the dentate gyrus. (A) Time course of synaptic plasticity experiments presented as a percentage of the baseline fEPSP. The black arrow indicates high-frequency stimulation (HFS) induction (4 × 100 Hz). Short-term plasticity is measured as the change in response size 1 minute following HFS. Long-term potentiation is measured as the change in response size 55 to 60 minutes following HFS. (B) Long-term potentiation is decreased following repetitive mild traumatic brain injury with sample traces (C) from sham and repetitive mild traumatic brain injury hippocampal slices. *P < 0.05 (Student’s *t*-test). Unpublished data. For more information on injury protocol, see Eyolfson and others (2021). For more information on electrophysiologic methods, see Fontaine and others (2019).

Studies examining synaptic plasticity in the hippocampus following TBI have generally employed moderate-to-severe TBI protocols. The literature on moderate-to-severe TBI demonstrates reductions in LTP following injury (Albensi and others 2000; D’Ambrosio and others 1998; Hung and others 2023; Norris and Scheff 2009; Reeves and others 1995; Schwarzbach and others 2006; Titus and others 2016; Yaka and others 2007; Zhang and others 2011). Of those investigating mTBI, most have used both in vivo and in vitro field electrophysiology in the CA1 regions of the HPC, leaving the DG understudied (Table 1). In CA1, deficits in LTP are seen between 2 days and 30 days postinjury. Although there is a high degree of variability in outcomes, more chronic time points (~PID30) with single injury models show no changes (McDaid and others 2021; White and others 2017), decreased LTP (Liu and others 2017), and increased LTP (Aungst and others 2014), and studies utilizing r-mTBI models also showing no changes (McDaid and others 2021) or decreased LTP (Aungst and others 2014; Sloley and others 2021; Zhang and others 2015). The degree of variability between studies may be associated with methodologic differences such as age at injury, model of injury, number of injuries, species used, and high-frequency stimulation protocol used. For example, following a single mTBI closed-head injury appears to produce no change in LTP, while fluid percussion injury results in decreased LTP. Following repetitive injuries, both open-head and closed- head injuries can produce prolonged deficits in LTP. As mentioned earlier, the DG is one of the only brain regions with ongoing neurogenesis and is highly susceptible to experience-dependent change (Gil-Mohapel and others 2013; Gil-Mohapel and others 2014; Olson and others 2006; Patten and others 2013; Yau and others 2014). To our knowledge, only one study has explored synaptic plasticity following a single mTBI in the DG. Using a weight-drop model of mTBI, White and colleagues found sex differences in LTP-dependent deficits that persisted for up to 28 days in the medial perforant pathway in females but not males (White and others 2017). Investigation of the effects of repetitive injuries in the dentate gyrus displays a similar deficit at seven days postinjury in females (Fig. 3).

**Table 1.** Hippocampal Synaptic Plasticity and Traumatic Brain Injury.

Article	Age	Sex; Strain	Model; Severity	Speed	Injuries	Sacrifice	Recording aCSF	Electrophysiology Technique	Short-Term Plasticity Findings	Long-Term Plasticity Findings
Hung and others (2023)	6–12 weeks	Male; C57Bl/6	CCI; moderate	2 m/s; 1 mm depth, 85 ms dwell	Single	14 days	117 NaCl; 4.5 KCl; 2.5 CaCl_2_; 1.2 MgCl_2_; 1.2 NaH_2_PO_4_; 25 NaHCO_3_; 11 glucose	DG (medial perforant pathway); LTP (TBS: 10 pulses, 5 bursts, 3 trains, 100 Hz)	Not reported	Decreased LTP
Hu and others (2022)	8–12 weeks	Female and male C57Bl/6	Closed-head injury; mild	3 m/s; 2.2 mm; 100 ms dwell	Repetitive (3–24 hours apart)	30 days	Standard aCSF not reported	CA1 (Schaffer-collateral); LTP (3 × 100 Hz)	Not reported	Decreased LTP
Lengel and others (2022)	P11	Female and male; Sprague-Dawley	CCI; moderate	5 m/s, 3 mm depth, 100 ms dwell	Single with hematomas	P48-60	126 NaCl; 26 NaHCO_3_; 2.5 KCl; 1.25 NaH_2_PO_4_; 5 MgCl_2_; 1 CaCl_2_; 10 glucose	CA1 (Schaffer collateral); LTP (TBS: 5 pulses; 4 trains; 100 Hz)	Measured at 10 minutes—reduced	Reduced LTP in TBI
McDaid and others (2021)	P60-80	Male; Long-Evans	Closed-head CCI; mild	6.5 m/s; 10 mm depth	Single and repetitive (3 injuries 48 hours apart)	30 days	125 NaCl; 2.5 KCl; 2 CaCl_2_; 1.2 MgSO_4_; 1.25 NaH_2_PO_4_; 25 NaHCO_3_; 10 D-dextrose	CA1 (Schaffer collateral); LTP (2 × 100 Hz)	No changes	No change in LTP in mTBI or r-mTBI
Sloley and others (2021)	6-12 weeks	Male; C57Bl/6	High-frequency head impact; mild	2.35 m/s; 7.5 mm depth	Repetitive (5 consecutive impacts/day for 6 days)	1 and 30 days	132 NaCl; 26 NaHCO_3_; 3 KCl; 1.25 NaH_2_PO_4_; 2 MgSO_4_; 2 CaCl_2_; 10 dextrose	CA1 (stratum radiatum); LTP (4 × 100 Hz)	Not reported	Reduced LTP at PID1 and PID30
Weston and others (2021)	3 months	Male; Sprague-Dawley	Lateral FPI; moderate	2.01 ± .05 atm	Single	30 and 60 days	124 NaCl; 3 KCl; 2 MgSO_4_; 26 NaHCO_3_; 1.25 NaH_2_P_4_; 2 CaCl_2_; 10 glucose	DG—medial perforant pathway; LTP (TBS—10 bursts; 4 pulses; 3 trains)	Not reported	No change between sham and 30 or 60 days; enhanced LTP at 60 compared to 30 days
Lecca and others (2019)	6–8 weeks	Male; CD1	Weight drop; mild	30 g/80 cm	Single	3 days	126 NaCl; 3 KCl; 2.4 CaCl_2_; 1.5 MgCl_2_; 1.2 NaH_2_PO_4_; 26 NaHCO_3_; 11 glucose	CA1 (Schaffer collateral); LTP (3 × 100)	Not reported	Decreased LTP
Liu and others (2017)	3 months	Male; C57Bl/6	Weight drop; mild	54 g/60 in.	Single	30 days	124 NaCl; 3 KCl; 1 MhCl_2_; 1.25 NaH_2_PO_4_; 2 CaCl_2_; 26 NaHCO_3_; 10 glucose	CA1 (Stratum radiatum); LTP (1 × 100 Hz)	Not reported	Decreased LTP
White and others (2017)	P25-28	Female and male; Long-Evans	Weight drop; mild	200 g weight from 10 cm	Single	1 hour; 1 day; 7 days; 28 days	125 NaCl, 2.5 KCL, 1.25 NaHPO_4_; 25 NaHCO_4_; 2 CaCl_2_; 1.3 MgCl_2_; 10 dextrose	DG (medial perforant pathway) and CA1 (Schaffer collateral); LTP (4 × 100 Hz)	Reduced STP in ipsilateral hemisphere in females at 7 and 28 days and males at 7 days	DG: Female: Ipsilateral—decreased LTP at 1, 7, 28 days; contralateral—no change Male: Ipsilateral—decreased LTP at 7 days; contralateral—no change; CA1—only difference in males at 7 days with decreased LTP
Furman and others (2016)	2 months	Male; Sprague-Dawley	CCI; moderate	3.5 m/s; depth 2 mm	Single	7 days	124 NaCl; 2 KCl; 1.25 KH_2_PO_4_; 2 MgSO_4_; 2 CaCL; 26 NaHCO_3_; 10 dextrose	CA1 (stratum radiatum); LTD (900 × 1 Hz)	Not reported	Enhanced LTD in ipsilateral hemisphere
Logue and others (2016)	7–8 weeks	Male; C57Bl/6	Closed-head CCI; mild	4 m/s; dwell time of 100 ms	Single and repetitive (3 injuries 24 hours apart)	7 days	119 NaCl; 2.5 KCl; 1 HaH_2_PO_4_; 11 D-glucose; 26.2 NaHCO_3_; 1.3 MgSO_4_*7H_2_O; 2.5 CaCl_2_	CA1 (Schaffer collateral); LTP (1 × 100 Hz)	Not reported	No difference in mTBI; enhanced LTP following r-mTBI in ipsilateral
Norris and others (2016)	Adult (250–275 g)	Male; Sprague-Dawley	CCI; moderate	Depth 2 mm, velocity 3.5 m/s	Single	7 and 14 days	124 NaCl; 2 KCl; 1.25 KH_2_PO_4_; 2 MgSO_4_; 2 CaCl; 26 NaHCO_3_; 10 dextrose	CA1 (stratum radiatum); LTD (1 Hz)	Not reported	PID7: Ipsilateral—increased LTD; contralateral—no change PID14: Ipsilateral—increased LTD; contralateral—no change
Titus and others (2016)	2–3 months	Male; Sprague-Dawley	FPI; moderate	2.0 atm	Single	3 months	125 NaCl; 2.5 KCl; 1.25 NaHPO_4_; 25 NaHCO_3_; 2 CaCl_2_; 1 MgCl_2_; 10 D-glucose	CA1 (Schaffer collateral); LTP (1 × 100 Hz)	Not reported	Decreased LTP
Almeida-Suhett and others (2015)	5–6 weeks	Male; Sprague-Dawley	Craniotomy CCI; mild	3.5 m/s; 200 ms dwell; 2 mm depth	Single	7 days	125 NaCl; 2.5 KCl; 2 CaCl_2_; 1.5 MgCl_2_; 25 NaHCO_3_; 1.25 NaH_2_PO_4_; 22 D-glucose	CA1 (Schaffer collateral); LTP protocol not mentioned	Not reported	Reduced LTP
Zhang and others (2015)	6–10 weeks	Male; C57Bl/6	Closed-head CCI; mild	3 m/s, depth 2.2 mm, dwell 100 ms	Repetitive (3 injuries 24 hours apart)	30 days	125 NcCl; 2.5 KCl; 1 MgCl_2_; 25 NaHCO_3_; 1.25 NaH_2_PO_4_; 2 CaCl_2_; 25 glucose; 3 pyruvic acid; 1 ascorbic acid	CA1 (Schaffer-collateral); LTP (TBS—10 bursts; 5 pulses; 3 trains; 100 Hz)	Not reported	Impaired LTP
Aungst and others (2014)	10–12 weeks	Male; Sprague-Dawley	Lateral FPI; mild	1.1 to 1.4 atm	Single or repetitive (3–48 hours apart)	28 days	125 NaCl; 2 KCl; 26 NaHCO_3_; 3 CaCl_2_; 1 MgCl_2_; 20 glucose	CA1 (Schaffer collateral); LTP (4 × 100 Hz)	Not reported	Single—no change contralateral; enhanced LTP ipsilateral Repetitive—impaired LTP contralateral and ipsilateral
Schwarzbach and others (2006)	5–7 weeks	Sex not specified; C57Bl/6	FPI; mild to moderate	1.4–2.1 atm	Single	7 days	126 NaCl; 3.2 KCl; 1.25 NaHPO_4_; 26 NaHCO_3_; 2 MgCl_2_; 2 CaCl_2_; 10 glucose	CA1 (Schaffer collateral); LTP—HFS (2 × 100 Hz) and TBS (5 × 100); LTD (1 Hz)	Not reported	Reduction in LTP for HFS and TBS; no change in LTD
Albensi and others (2000)	Young adult (150–300 g)	Male; Sprague-Dawley	CCI; moderate	Not mentioned	Single	2 days	124 NaCl; 25 KCl; 1.24 NaH_2_PO_4_; 1.3 MgSO_4_; 2.4 CaCl_2_; 26 NaHCO_3_; 10 glucose	CA1—LTD (1 Hz); LTP (100 Hz)	Not reported	Population spike analysis; LTD—enhanced LTP—impaired
D’Ambrosio and others (1998)	P24-31	Male; Sprague-Dawley	FPI; moderate to severe	3.5 atm	Single	1–2 days	120 NaCl; 3.1 KCl; 1 MgCl_2_; 1.25 KH_2_PO_4_; 26 NaHCO_3_; 2 CaCl_2_; 10 dextrose	CA1 (Schaffer collateral); LTP (2 × 100 Hz); LTD (1 Hz)	Not reported	LTP—reduced LTD—no change
Sick and others (1998)	250–300 g	Male; Sprague-Dawley	FPI; mild to moderate	1.7–2.1 atm	Single	4 and 48 hours	126 NaCl; 3.5 KCl; 2 CaCl_2_; 2 MgSO_4_; 26 NaHCO_3_; 1.25 NaH_2_PO_4_; 10 glucose	CA1 (Schaffer collateral); LTP (100 or 200 Hz)	Not reported	Reduced LTP at 4 and 48 hours

NOTE: All results are comparisons between nontreated shams and nontreated TBIs where applicable.

aCSF = artifical cerebral spinal fluid; CCI = controlled cortical impact; DG = dentate gyrus; FPI = fluid percussion injury; HFS = high-frequency stimulation; mTBI = mild traumatic brain injury; PID = post-injury day; r-mTBI = repetitive mild traumatic brain injury; STP = short-term potentiation; TBI = traumatic brain injury; TBS = theta burst stimulation.

While deficits in LTP are well documented, there is still much to uncover regarding LTD. Albensi and others (2000) found enhanced LTD 48 hours following moderate-to-severe TBI. In contrast, others have found no change in the induction or maintenance of LTD post-TBI (D’Ambrosio and others 1998). Other models of moderate TBI have seen enhanced LTD up to two weeks postinjury (Albensi and others 2000; D’Ambrosio and others 1998; Furman and others 2016; Norris and others 2016; Schwarzbach and others 2006; Zhang and others 2018). Interestingly, age at injury could be a determining factor for altered LTD, particularly with a 900 × 1-Hz protocol. Studies that have used juvenile or early adolescent animals have not identified differences in LTD, while other studies using late adolescent or young adult animals observe enhanced LTD following injury. Kemp and colleagues investigated this relationship between age and low-frequency stimulation for the induction of LTD. The researchers found that induction of a 900 × 1-Hz protocol induced LTD in neonatal (P12–20) and early adolescent (P31–40) animals. However, 900 × 1 Hz did not induce LTD in middle adolescence (P40–50) or adulthood (12–16 weeks) (Kemp and others 2000). Future research is imperative to elucidate the direct relationship between changes in synaptic plasticity and TBI-induced cognitive deficits as aberrant LTD has been associated with impaired spatial memory consolidation (Ge and others 2010).

### Endocannabinoids and Synaptic Plasticity

The eCB system is important for the normal functioning of the HPC, as CB1Rs are found on GABAergic and glutamatergic neurons (Fontaine and others 2020). Due to their location on primary excitatory and inhibitory neurons, eCBs may modulate the excitatory/inhibitory balance under homeostatic conditions. Of importance, eCBs can mediate LTD through retrograde signaling. Previous studies from our laboratory have highlighted the involvement of the eCBs in LTD and demonstrated that LTD can be blocked by the administration of a CB1 inverse agonist (Fontaine and others 2020; Peñasco and others 2019). The role of CB1Rs on the presynaptic terminal of both GABAergic and glutamatergic neurons suggests they are a determining factor in all aspects of synaptic plasticity (Wang and others 2016). Wang and colleagues found that perfusion of AM251 (a CB1R inverse agonist) before HFS, or the use of CB1R knockout mice, resulted in severely attenuated LTP in the lateral perforant pathway of the DG. Additionally, inhibition of monoacylglycerol lipase (MGL), thereby increasing levels of 2-AG, enhanced LTP in the lateral perforant pathway (Wang and others 2016). Based on this study, there appear to be regional differences in the HPC, as perfusion of AM251 did not attenuate LTP in the medial perforant or Schaffer collateral pathways. However, the authors used different HFS protocols in each of the three pathways. It is unclear if the use of the same paradigm (3 × 100 Hz) across all pathways would have been sufficient to see deficits in LTP with AM251.

The role of eCBs in LTP depends on the specific HFS protocol administered and the presence of exogenous cannabinoids. In Schaffer collateral fibers, weak theta burst stimulation (TBS; 5 trains, 100 Hz, four stimuli, 200 ms) with AM251 enhances LTP while strong TBS (10 trains, 100 Hz, four stimuli, 200 ms) with AM251 suppresses LTP (Silva-Cruz and others 2017). Additionally, the continuous activation of CB1R with the administration of an eCB receptor agonist attenuates LTP (which can be recovered by coadministration of AM251) (Silva-Cruz and others 2017). In support of this result, Le and others (2022) injected 5 mg/kg (intraperitoneal) THC into female and male mice (C57Bl/6) and rats (Long Evans) for two weeks. Field electrophysiologic recordings were conducted in adulthood, and there were strain-, sex-, and region-dependent changes in LTP. In CA1 neurons, females, but not males, displayed reductions in LTP, while reductions were observed in both females and males in the lateral perforant pathway (Le and others 2022). Thus, exogenously activating the endocannabinoid system has long-term consequences for adult behavior and synaptic plasticity.

### Endocannabinoids and TBI

The role of eCBs following TBI may depend on a variety of factors, including the localization of CB1Rs and CB2Rs. Endogenous CBs may be synthesized “on demand” to help regulate excitotoxicity that can occur following a TBI. In a mouse model of excitotoxin-induced injury in the HPC, anandamide levels increased, and activation of CB1 receptors was necessary for neuronal survival (Marsicano and others 2003). CB1 receptors are found on both pre- and postsynapses, as well as on astrocytes, making it unclear which mechanism is more prominent in excitotoxin-induced injury. Research into similar neuropathologic diseases that also exhibit excitotoxicity, such as multiple sclerosis, Parkinson disease, and Alzheimer disease, may aid in understanding the pathology of mTBI. For example, in a mouse model of multiple sclerosis (MS), the activation of CB1Rs and CB2Rs results in neuroprotective effects against glutamate excitotoxicity (Loría and others 2010), implying that CB1 exerts retrograde inhibition of glutamate excitotoxicity. This suggests that eCBs could be trialed as a therapeutic strategy to alleviate glutamate excitotoxicity associated with the secondary injury cascades that accompany TBI pathology.

Following TBI, glutamate excitotoxicity stimulates the production of 2-AG, which, via CB2 signaling, recruits microglia to the site of injury (Walter and others 2003). Following all severities of TBI (including r-mTBI), there is time-dependent infiltration of peripheral immune cells into the brain (Eyolfson and others 2020). The mechanism for this is not yet understood, but given that macrophages also express CB2Rs and play roles in the inflammatory profile postinjury, this is important for understanding the interplay between endocannabinoid signaling, microglia, and macrophages as a therapeutic target for modulating post-TBI inflammation. In an APP/PS1 mouse model of Alzheimer disease, mice lacking CB2R displayed lower levels of proinflammatory cytokines and reductions in infiltrating macrophages (Schmöle and others 2015). However, establishing a direct connection to mTBI is not straightforward, as reductions in cytokine levels during the acute postinjury period have beneficial or detrimental effects in other TBI pathologies. For instance, in TNFα knockout mice, moderate-to-severe TBI resulted in worse behavioral and histologic outcomes when compared to wild types (Scherbel and others 1999). Conversely, the ablation of Il-1R in adult male mice reduced proinflammation cytokine expression and enhanced cognitive functioning, following fluid percussion injury (Newell and others 2018).

Research on the influence of eCBs and TBI has ignored one of the most at-risk populations: adolescents with mTBI. This is critical as adolescents have among the highest rates of cannabis use. Cannabis is the most commonly used illicit drug among high school students, with 11% reporting previous 30-day use and 3.1% reporting daily use (Johnston and others 2022). Furthermore, given the heightened risk for mTBI and the use of cannabis during adolescence, it is possible that this pharmacologic exposure could exacerbate or alleviate symptoms. We are aware of one study that has used a closed-head model of r-mTBI and repeated exposure to cannabis either before or postinjury. Bhatt and colleagues (2020) examined the therapeutic and neuroprotective effects of injected THC (1.25 mg/kg) in two experiments. In experiment 1, adolescent rats received three closed-head injuries with the lateral impact model of mTBI and 12 daily injections of THC following the final injury. The authors reported that treatment of THC alleviated anxiety-like and depressive-like behaviors, as well as deficits in working memory. Additionally, in the hippocampus, treatment with THC alleviated injury-induced decreases in the cannabinoid receptor 1 gene (a coding gene for CB1 receptors). In experiment 2, the authors examined the neuroprotective potential of THC but found that preinjury injections of THC did not improve behavioral outcomes (Bhatt and others 2020). This series of experiments provides evidence that THC may have therapeutic potential following injury but not as a preemptive measure. It is important to note that as administration of THC in these experiments was at sub-physiologic levels, researchers will need to examine these effects with ecologically valid methods of THC administration (i.e., inhalation) and the effects of higher dosages (5–30 mg/kg).

### 2-AG and AEA

In general, expression of 2-AG and AEA is enhanced in response to TBI of all severities (Xu and others 2019). Since eCBs are produced “on demand,” it is possible that the CNS synthesizes them as a compensatory neuroprotective mechanism post-TBI (Panikashvili and others 2001). Following a single moderate-to-severe TBI, levels of AEA increased in the ipsilateral cortex but not the contralateral cortex, while no changes were seen in 2-AG at three days postinjury. When TBI animals were treated with a fatty acid amide hydrolase inhibitor, levels of AEA increased in the ipsilateral and contralateral cortex while levels of 2-AG were only increased in the ipsilateral cortex. Increased eCB expression was correlated with improved recovery in anxiety, working memory, and motor coordination (Tchantchou and others 2014). The same research group demonstrated similar effects with a closed-head r-mTBI model. Selvaraj and others (2021) found that following r-mTBI, there were no changes in AEA or 2-AG in the ipsilateral cortex on postinjury day 4. Yet, treatment (once per day, for 8 days, beginning 5 days following the final injury) with an FAAH inhibitor increased expression of AEA, but not 2-AG, and attenuated motor and working memory performance up to two weeks postinjury. However, it does not appear that the researchers included sham mice that were injected with the FAAH inhibitor. To our knowledge, only one study has included chronic time points. Following moderate-to-severe TBI, reduced subcortical concentrations of AEA up to 155 days postinjury were identified (Vogel and others 2020). However, this study did not see any changes in 2-AG expression during this chronic time point.

Research suggests that inhibiting 2-AG degradation may also improve TBI-induced behavioral outcomes and attenuate excitatory postsynaptic potentials. Following lateral fluid percussion mTBI in adult male mice, inhibition of 2-AG degradation improved behavioral outcomes and attenuated miniature excitatory postsynaptic potentials in the ipsilateral cortex (Mayeux and others 2017). This research group also demonstrated that under the same conditions, similar behavioral improvements could be linked to inhibition of 2-AG degradation, but there were no changes in *IL-1B, Il6, CCl2, TNF-a, COX2*, or *NOX2* mRNA expression (Katz and others 2015). Additionally, following the inhibition of a 2-AG inhibiting enzyme (monoacylglycerol lipase), adult male mice displayed elevated levels of 2-AG, diminished behavioral deficits, and reduced inflammatory profiles following closed-head controlled cortical impact (CCI) r-mTBI (Selvaraj and others 2021). It is currently unclear whether 2-AG or AEA is more important (or what their relationship is following injury) as there exists high regional variability in their expression (Buczynski and Parsons 2010). Thus, eCBs have the potential to attenuate secondary injury cascades in acute and chronic timeframes postinjury.

### CB1 and CB2 Receptors

**Figure 4.**
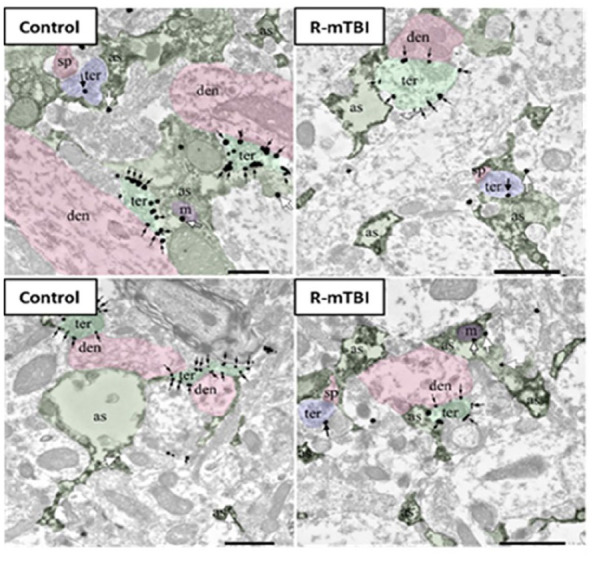
CB1R immunolocalization in the molecular layer of the dentate gyrus. Electron microscopy of CB1R immunogold particles (black and white arrows) is localized to inhibitory (ter, light green) and excitatory synaptic terminals (ter, blue) as well as to astrocytes (as, dark green) and mitochondria (m, purple). Postsynaptic dendrites (den, pink) and dendritic spines (sp, pink) are CB1R immunonegative. For more information on electron microscopy methodology, see Bonilla-Del Río and others (2021).

Most studies investigating the levels of CB1 or CB2 receptors following TBI are done in the context of moderate-to-severe injury. Interestingly, these two primary eCB receptors exhibit dichotomous responses. In the acute period following moderate-to-severe TBI, *CB2R* mRNA increased in the ipsilateral cortex while *CB1R* mRNA expression decreased or showed no change (Braun and others 2018). The majority of studies failed to distinguish between discrete brain regions, but the same trends appeared in the cortex, striatum, and HPC (Song and others 2021). In the DG, electron microscopy images of CB1R expression and density suggest significant decreases in inhibitory and excitatory terminals (Fig. 4). Many studies have attempted to use CB1R and CB2R receptor agonists and antagonists to examine changes in TBI pathophysiology. The specific aims of these studies are diverse but focus on the neuroinflammatory response. Overall, the use of CB2R agonists (GP1) improves behavioral outcomes and reduces inflammatory profiles in the acute time frame following moderate-to-severe TBI (Amenta and others 2012; Amenta and others 2014; Braun and others 2018), while the use of antagonists (AM630; JWH-133) worsens behavioral and neuroinflammatory profiles (Amenta and others 2012; Amenta and others 2014; Braun and others 2018).

Contradictory results between the expression of receptors and behavioral deficits were identified, with reduced CB1 expression resulting in worse behavioral deficits and increased CB2 receptor expression also leading to worse behavioral deficits (Lopez-Rodriguez and others 2015a). However, the use of CB1 and CB2 receptor antagonists did not significantly improve outcomes following moderate-to-severe TBI (Lopez-Rodriguez and others 2015b). The results in young adult males are similar to those in aged males (5–6 months). Within the acute period following moderate-to-severe TBI, there was increased expression of CB2R but not CB1R in the ipsilateral cortex. However, in aged animals, this effect reversed at 28 days postinjury (increased CB1R but not CB2R) (Ahluwalia and others 2023). Following moderate-to-severe TBI in eight-week-old male mice and postinjury treatment with CB2 receptor agonist (0-1966A), the authors demonstrated improved motor performance on the rotarod and cylinder tests. Additionally, there was reduced expression of Iba1^+^ microglia in the ipsilateral hemisphere (Elliott and others 2011). Few studies have investigated the use of CB1 and CB2 agonists following mTBI. When a CB1R antagonist (AM281) was administered following a single weight-drop mTBI in adult (P90) male mice, elevated levels of 2-AG levels in the HPC were identified five days postinjury. Additionally, the use of a CB1 agonist (AM281) rescued TBI-induced deficits in spatial navigation (Xu and others 2019). Further research is needed to fully understand the role of eCBs in the brain following injury and to determine whether they can be used as a therapeutic target.

## Endocannabinoids as a Therapeutic Avenue for mTBI-Induced Deficits in Synaptic Plasticity

**Figure 5.**
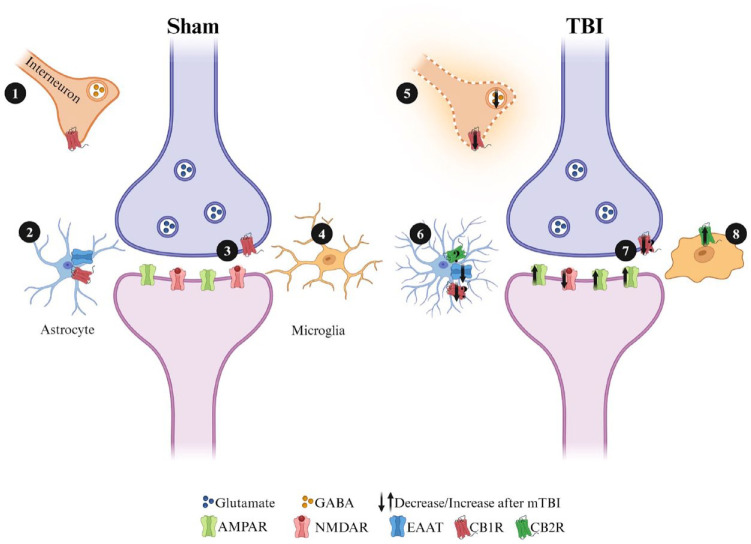
Simplified graphic displaying the potential roles CB1 receptors could play in synaptic plasticity at excitatory hippocampal synapses. Cannabinoid receptors are located in a number of places where they have the potential to significantly affect synaptic plasticity. In response to injury, microglia are activated and proliferate as part of the proinflammatory response. Astrocytes also contribute to neuroinflammation by becoming reactive following injury. Both astrocytes and microglia synthesize endocannabinoids (eCBs) and express cannabinoid receptors (CBRs), which could potentially aid in decreasing neuroinflammation. (1/5) CB1 receptors located on presynaptic terminals that release GABA can reduce inhibitory tone when activated. This can lead to greater postsynaptic excitation and enhance the capacity for LTP at excitatory synapses. Following mild traumatic brain injury (mTBI), glutamate excitotoxicity results in decreased GABAergic functioning and interneuron cell death, further driving aberrant excitatory transmission. (2/6) CB1R located at astrocytes can reduce the uptake of glutamate, both increasing excitatory drive at a synapse and lowering the conversion of glutamate to GABA by astrocytes. Following injury, upregulation of CB2R on astrocytes can reduce proinflammatory responses and improve conditions for neuronal survival. (3/7) Retrograde transmission of eCBs synthesized from the postsynaptic terminal can interact with CB1Rs located on presynaptic terminals and modulate the release of glutamate. (4/8) CB2Rs located on microglia are downregulated under homeostatic conditions, and following mTBI, CB2R is upregulated to reduce proinflammatory responses.

Over the past decades, the concept of the synapse has evolved to include the astrocytes that surround pre- and postsynaptic elements as part of a functional unit referred to as the “tripartite” synapse (Araque and others 1999; Gutiérrez-Rodríguez and others 2018). There is also evidence that other populations of glial cells, including NG2 glia and microglia, can also play critical roles in synaptic function and synaptic plasticity (Dityatev and Rusakov 2011). We hypothesize that eCBs may play a significant role in synaptic plasticity deficits following injury, given their widespread expression throughout the synaptic cleft (Fig. 5). Deficits in synaptic plasticity are unlikely to be attributable solely to changes in eCB ligands or receptor expression at any one specific location but rather result from the combination of alterations in inhibitory and excitatory neurotransmitter signaling, changes in expression in astrocytes and microglia, and other factors yet to be determined. eCBs are synthesized “on demand” by postsynaptic neurons and glial cells following injury as an attempt to counteract glutamate excitotoxicity and mitigate rampant proinflammatory responses. Based on the results from Silva-Cruz and colleagues (2017), the role of eCBs may hinge on the strength of HFS protocols, where strong HFS is eCB dependent and weak HFS is eCB independent. To actually resolve this question, additional research is imperative, given most studies exploring LTP following mTBI have used a strong HFS protocol (any number of pulses at 100 Hz; Table 1). The use of a monoacylglycerol lipase (MAGL; 2-AG degrading enzyme) inhibitor, thereby increasing levels of 2-AG, demonstrates a neuroprotective effect on the neuroinflammatory profile, synaptic plasticity, and learning and memory following TBI (Hu and others 2022; Katz and others 2015; Mayeux and others 2017; Selvaraj and others 2021; Zhang and others 2015). The intersection of eCBs, mTBI, and their influence on synaptic plasticity has often been overlooked. We are aware of one study that inhibited MAGL with seven injections of JZl184 (10 mg/kg—30 minutes after each injury and up to PID4). Interestingly, inhibition of MAGL rescued impaired LTP in Schaffer collateral synapses on PID30 (Zhang and others 2015). It is possible that inactivation of MAGL could be reducing proinflammatory markers through peroxisome proliferator-activated receptors (PPARs), as evidenced by PPAR’s ability to alleviate inflammation following moderate-to-severe TBI (Wang and others 2020). PPAR activation has also been shown to increase GluR1 AMPAR expression and rescue LTP deficits in a mouse model of cognitive impairment (Pierrot and others 2019). Yet, more research is required to better understand how single and repetitive mTBI impact CB receptor distributions, as well as the capacity for endogenous ligands to play a role in their activation.

The eCBs present a promising avenue for therapeutic intervention post-TBI as eCBs have been shown to promote gliogenesis and neurogenesis, as well as reduce proinflammatory cytokine levels (Hill and others 2010; Jin and others 2004; Walter and others 2003). Given these functions and the distribution of cannabinoid receptors, we cannot downplay the regulatory processes they may invoke in astrocytes and microglia. Glutamate excitotoxicity is a prominent feature identified across all severities of TBI and manifests in numerous ways, including via changes in receptors such as glutamate transporter 1 (GLT1) and excitatory amino acid transporters (EAATs) on astrocytes (for a comprehensive review on receptor changes following TBI, see Guerriero and others 2015). The modulation of astrocytic receptors is crucial for TBI pathology, as astrocytes play a pivotal role in glutamate reuptake. Consequently, a decrease in the expression of GLT-1 and EAAT leads to a decrease in glutamate reuptake and subsequent conversion to glutamine and GABA. Over time, this aberrant functioning results in GABAergic interneuron cell death and persistent GABA-A receptor dysfunction, ultimately contributing to hyperexcitability (Becerra and others 2021; Zhang and others 2007). This dysfunction is thought to underpin cognitive deficits following TBI. A recent study investigated genetic knockouts for MAGL in both astrocytes and neurons following r-mTBI in mice. The knockout of MAGL attenuated the increase in expression of IBA1^+^ and GFAP^+^ microglia and astrocytes in the cortex, CA1, and DG while also preventing axonal injury and degeneration. Additionally, MAGL inactivation rescued cognitive deficits in the Morris Water Maze (Hu and others 2022). Moreover, the use of a CB1R agonist rescued injury-induced deficits in working and spatial memory following moderate-to-severe TBI (Arain and others 2015). Taken together, these findings suggest that eCB activation following injury can ameliorate TBI-induced memory deficits, both acutely and persistently. Notably, behavioral testing in these experiments occurred three weeks following treatment, underscoring the sustained impact of eCB-based interventions on cognitive outcomes.

## Conclusion

This review did not go into detail about the effects of sex on eCB, synaptic plasticity, and mTBI, but that does not negate its importance. The primary issue is that the majority of discovery research has been conducted with male subjects (Bodnar and others 2019; Mamlouk and others 2020). For example, of the studies using mTBI and measuring synaptic plasticity, to our knowledge, only one has included female animals for LTP and none for LTD. In eCBs, current research has shown negligible sex differences in CB1 receptor expression in the prefrontal cortex (PFC), striatum, and HPC. However, there may be differences in receptor activation, as a recent study reported enhanced activation of CB1 receptors in the HPC of female rats (Farquhar and others 2019). Additionally, chronic THC exposure results in more pronounced CB1 receptor downregulation in females (Farquhar and others 2019). Finally, route of administration and THC metabolite differences between females and males may be important areas necessitating investigation (Baglot and others 2021). Expression of THC’s metabolite, 11-hydroxy-THC, is enhanced in females compared to males but only if injected (compared to inhalation). Increased metabolism to 11-hydroxy-THC is thought to underpin sex differences in behavior following THC administration (Tseng and others 2004). This effect may not be exclusively attributed to exogenous cannabinoids as increased anandamide and 2-AG signaling altered extinction behaviors in females but not males (Morena and others 2021). As we navigate the complex interplay between eCBs, TBI, and synaptic plasticity, it will also be important to further explore the role of sex differences in the eCB system, both for normal functioning and in the aftermath of TBI, to truly understand the therapeutic potential of this system.

There is still a paucity of data for the cognitive deficits that can appear following mTBI. These deficits may reflect impairments in hippocampal functioning and likely involve changes in the capacity of the system to express and maintain bidirectional synaptic plasticity (i.e., both LTP and LTD). Our understanding of these deficits necessitates comprehension of the time course of changes in synaptic plasticity following TBI, as well as how TBI impacts CB receptor expression. This area of research requires a comprehensive investigation that will include the use of transgenic animal models, pharmacologic agonists and antagonists of CB receptors, and RNA sequencing to fully elucidate a potential mechanism of action. The findings presented here underscore the significance of considering diverse factors, such as specific HFS protocols, regional variations in plasticity, and the involvement of eCBs. We hypothesize that the eCBs present potential therapeutic avenues. They not only provide insights into synaptic plasticity but also suggest promising directions for future research and clinical interventions.
